# Subclinical Atherosclerosis and Cardiovascular Events Among Patients With Colorectal Cancer

**DOI:** 10.1002/cam4.70938

**Published:** 2025-05-14

**Authors:** Julia A. Levy, Elham Kazemian, Cody Ramin, Nicole C. Loroña, Maimoona Nadri, Jordan O. Gasho, Katrina D. Silos, Andriana P. Nikolova, Damini Dey, Erin M. Siegel, Biljana Gigic, Sheetal Hardikar, Doratha A. Byrd, Adetunji T. Toriola, Jennifer Ose, Christopher I. Li, David Shibata, Cornelia M. Ulrich, Balaji K. Tamarappoo, Katelyn M. Atkins, Jane C. Figueiredo

**Affiliations:** ^1^ Department of Medicine Samuel Oschin Comprehensive Cancer Institute, Cedars‐Sinai Medical Center Los Angeles California USA; ^2^ Department of Internal Medicine Oregon Health & Science University Portland Oregon USA; ^3^ Department of Computational Biomedicine Cedars‐Sinai Medical Center Los Angeles California USA; ^4^ Department of Radiation Oncology Samuel Oschin Comprehensive Cancer Institute, Cedars‐Sinai Medical Center Los Angeles California USA; ^5^ Department of Cardiology Cedars‐Sinai Medical Center Los Angeles California USA; ^6^ Department of Biomedical Sciences Cedars‐Sinai Medical Center Los Angeles California USA; ^7^ Department of Cancer Epidemiology Moffitt Cancer Center Tampa Florida USA; ^8^ Epidemiology and Genomics Program, Division of Cancer Control & Population Sciences National Cancer Institute Washington DC USA; ^9^ Department of General, Visceral and Transplantation Surgery Heidelberg University Hospital Heidelberg Germany; ^10^ Department of Population Health Sciences University of Utah Salt Lake City Utah USA; ^11^ Division of Public Health Sciences, Department of Surgery Washington University School of Medicine St. Louis Missouri USA; ^12^ University of Applied Sciences and Arts Department of Information and Communication Hanover Germany; ^13^ Division of Public Health Sciences Fred Hutchinson Cancer Research Center Seattle Washington USA; ^14^ Department of Surgery University of Tennessee Health Science Center Memphis Tennessee USA; ^15^ Department of Cardiovascular Medicine Mayo Clinic Scottsdale Arizona USA

**Keywords:** atherosclerosis, colorectal cancer, coronary artery calcium

## Abstract

**Background:**

Prior studies have documented that patients with colorectal cancer (CRC) are at an increased risk of cardiovascular disease (CVD).

**Objectives:**

To examine coronary artery calcium (CAC) as a marker of subclinical atherosclerosis and its association with major adverse cardiovascular events (MACE) in patients with CRC across the cancer treatment trajectory.

**Methods:**

Adults with newly diagnosed CRC were enrolled in the prospective ColoCare study from 2017 to 2024. CAC was measured from routine diagnostic computed tomography (CT) and positron emission tomography‐CT scans at CRC diagnosis until 5 years post‐diagnosis. Atherosclerosis was defined as the presence of CAC. We used multivariable‐adjusted Fine and Gray models to assess the association between CAC and MACE risk, accounting for competing risks.

**Results:**

Among 300 CRC patients, the most common CVD risk factors at cancer diagnosis were hypertension (37%), hyperlipidemia (24%), and diabetes (14%). During follow‐up (median = 5.3 years), 75 (25%) individuals experienced MACE: stroke (3%), new/worsening HF (9%), HF exacerbation requiring hospitalization (2%), coronary revascularization (3%), and death (19%). Among individuals with imaging at baseline (*n* = 101), 37 (36.6%) had CAC, and statins were not prescribed in 11 (55.0%) patients with moderate/high CAC. For those with serial imaging (*n* = 61), 31.1% showed worsening CAC and 3% developed new CAC. Baseline CAC conferred a higher risk of MACE (HR = 4.79; 95% CI: 1.05–21.75, *p* = 0.04) after accounting for cancer‐related deaths as a competing risk.

**Conclusions:**

Subclinical atherosclerosis and MACE are common among patients with CRC. Integrating CAC from routine cancer imaging can identify patients who may benefit from cardio‐preventive treatment.

## Graphical abstract

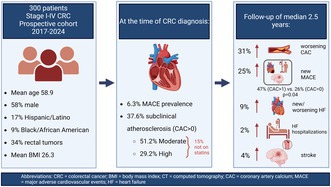

## 1 Introduction

Colorectal cancer (CRC) is the third most common cancer worldwide and the second most common cause of cancer‐related death [1]. In 2022, the estimated number of CRC survivors grew to approximately 1.4 million, largely due to advances in early detection and improved cancer therapies [2]. While this trajectory is encouraging, patients with CRC may have a higher than average risk of cardiovascular disease (CVD) [3, 4, 5, 6, 7, 8, 9, 10, 11], the leading cause of mortality worldwide [12]. Shared etiologic risk factors, such as age, smoking, obesity, alcohol consumption, dietary factors, and physical inactivity, are thought to partially explain the link between CRC and CVD [13, 14, 15, 16, 17, 18]. Furthermore, cancer treatment modalities including chemotherapy and immunotherapy can be cardiotoxic [19, 20, 21, 22], heightening the risk of CVD in these vulnerable patients who often receive multimodality treatment.

A few studies have documented the prevalence of CVD risk factors in patients diagnosed with CRC. These studies have observed that coronary artery disease and hypertension were among the most common comorbid conditions in newly diagnosed CRC patients and that such conditions were associated with reduced survival [23, 24, 25]. Some studies have reported that CRC patients are at increased risk of new‐onset CVD after the diagnosis of CRC (especially within the first 3 years) as compared to cancer‐free controls [3, 5, 21, 26]. Therefore, predictors of susceptibility around the time of a cancer diagnosis could help identify high‐risk individuals. Subclinical atherosclerosis, characterized by the asymptomatic presence of atherosclerotic plaque, has been increasingly recognized as an important marker of cardiovascular risk. Preexisting subclinical atherosclerosis can be detected by the presence of coronary artery calcification (CAC) from computed tomographic (CT) imaging of the chest, which is routinely performed for staging and surveillance in most CRC patients. CAC is recognized as a robust, validated marker of risk for major adverse cardiac events (MACE) [27, 28]. Among cancer patients, CAC is associated with both an increase in cardiac‐ and cancer‐related mortality in patients with various cancer types [29, 30, 31, 32, 33, 34, 35]. However, the prevalence of atherosclerosis in CRC patients without clinical symptoms of CVD has not been well characterized.

As a result of this limited knowledge to date, current guidelines for CVD assessment in CRC patients are undefined [36, 37]. Furthermore, traditional CVD risk tools, such as the atherosclerotic cardiovascular disease (ASCVD) risk score, have not been validated in cancer populations in whom malignancy and cardiotoxic cancer treatment may enhance the risk of MACE. Our study examined CAC at the time of CRC diagnosis, during treatment, and into survivorship.

## 2 Methods

### 2.1 Study Design and Participants

This study used data from a prospective cohort, the international ColoCare Study (ClinicalTrials.gov identifier: NCT02328677) [38]. The ColoCare Study includes men and women aged 18–89 who were newly diagnosed with Stage I–IV colon, rectum, or rectosigmoid cancer prior to surgery at seven sites throughout the United States and Germany. In this analysis, patients were all recruited in the clinical setting at a large healthcare system in Southern California, from 2017 to 2024. All participants provided written informed consent before any study procedures took place. The ColoCare Study was approved by the Institutional Review Board at Cedars‐Sinai Medical Center.

### 2.2 Patient‐Reported Data

Self‐administered questionnaires were sent electronically to patients at the time of enrollment (baseline), 6 months after enrollment, and annually thereafter. Surveys included questions regarding demographics, medications and nutritional supplement use, risk factors (e.g., smoking, alcohol intake, physical activity), and clinical histories.

### 2.3 Clinical Data Abstraction

Electronic medical records (EMRs) were reviewed according to a standardized process to extract demographic information and clinical data including medical comorbidities, medications, basic labs, tumor details, and anticancer treatment regimens. Relevant comorbidities identified through provider documentation included history of hypertension, hyperlipidemia, diabetes mellitus (DM), arrhythmias, myocardial infarction (MI), percutaneous coronary intervention (PCI), coronary artery bypass graft (CABG), stroke, thromboembolism, valvular heart disease, peripheral arterial disease (PAD), chronic kidney disease (CKD), and prior malignancies. Tumor characteristics (date of diagnosis, stage, site) were abstracted from pathology reports, imaging data, and provider documentation. Laboratory data of interest included lipid panel, complete blood count (CBC), comprehensive metabolic panel (CMP), and cardiac biomarkers. Cancer treatment regimens, adjuvant and neoadjuvant chemotherapy, radiation, and surgical resection(s), were identified through provider documentation. The timing of CRC treatment administration was identified relative to MACE.

### 2.4 CAC Assessment

Diagnostic images at baseline were available in a subset of patients (*n* = 101; Figure S1). CAC was measured manually on these routine CT and PET‐CT scans using syngo.via (Siemens Healthineers, Malvern PA). All scans were non‐ECG gated and noncontrast, with 2.5–5.0 mm slice thickness. Follow‐up images were identified as occurring at least 1 year after their first oncologist appointment. Images were excluded if they did not include the entire heart or if there was evidence of significant motion artifact. CAC was measured using the Agatston method in each of four coronary structures (left main artery, left anterior descending, left circumflex, and right coronary artery) and subsequently summed to find the total CAC score, total number of plaques, and total calcium volume. CAC > 0 was defined as the presence of subclinical atherosclerosis. We constructed risk groups defined as none (0), mild (1–99), and elevated (≥ 100) [39]. Change in CAC was calculated as the difference between baseline and postdiagnosis total CAC scores.

### 2.5 Adiposity Measures

Body mass index was abstracted from medical records at the time of diagnosis. Epicardial fat was quantified using semiautomated QFAT2.0 postprocessing software using the technique outlined by Commandeur et al. [40]. In brief, we measured epicardial adipose fat around the heart from the pulmonary trunk bifurcation to the inferior heart surface as defined by a standard fat attenuation range in Hounsfield units. The Syngo.via software was used to measure visceral and subcutaneous fat in three contiguous slices centered at the third lumbar vertebra (L3). Within the abdominal cavity, pixels containing subcutaneous adipose tissue were identified in Hounsfield unit values ranging between −190 and −30, abdominal muscle ranging from −29 to −150, and visceral adipose tissue ranging from −150 to −50. Each tissue was then selected using an automated dual threshold segmentation function and isolated via manual contour.

### 2.6 Cardiac Variables

The primary cardiac outcomes were the incidence of MACE between the CRC diagnosis date and the end of follow‐up (March 31, 2024). MACE was defined as stroke, heart failure (HF) exacerbation requiring hospitalization, new or worsening HF, acute MI, coronary revascularization (defined as CABG or PCI), and all mortality [41]. Transthoracic echocardiograms (TTE) were used to identify new HF (defined as new systolic or diastolic dysfunction as reported on TTE) and worsening HF (defined as an absolute decline ≥ 5% from left ventricular ejection fraction from baseline TTE). Baseline and repeat TTEs were defined as studies performed before and after the diagnosis of CRC, respectively. Other cardiometabolic outcomes of interest included new or worsening hypertension or hyperlipidemia (defined as requiring medication changes). Data on the above events were identified through ICD‐10 codes that were then confirmed by standardized manual chart review.

### 2.7 Risk Score Calculation

We conducted a risk assessment among patients using the ASCVD score to calculate each patient's 10‐year risk of ASCVD in patients without a known history of CVD. The ASCVD score incorporates various cardiovascular risk factors such as age, sex, race, total cholesterol, high‐density lipoprotein cholesterol, systolic blood pressure, use of antihypertensive medication, diabetes status, and smoking history. Clinical data abstraction as described above was performed to acquire the necessary data for ASCVD score calculation. Both former and current smokers constituted a positive smoking history. The blood pressure values and lipid values from routine laboratory tests available closest to the time of cancer diagnosis were used for calculation. To address missing data for ASCVD risk score, we used the Multiple Imputation by Chained Equations (MICE) method. Predictive Mean Matching (PMM) was applied as the imputation technique, generating five imputed datasets to enhance the robustness of the estimates [42].

### 2.8 Statistical Analysis

The baseline characteristics by CAC score were compared using Fisher's exact or chi‐squared test for categorical variables, and independent samples t‐tests for continuous variables. To compare CAC categories between the first and last scans, we used McNemar's test to evaluate significant changes in each category over time. Cox proportional hazards models were used to examine the association between CAC and MACE and between CAC and all‐cause mortality. To account for competing risks of cancer‐related deaths, we used the Fine and Gray subdistribution hazard models [43]. Hazard ratios (HRs) with 95% confidence intervals (CIs) were estimated with the CAC = 0 group as the reference. Covariates were selected based on their clinical relevance to the association between CAC and MACE, as identified through the literature. Final models were adjusted for age (continuous), sex, tumor site (colon vs. rectal), chemotherapy treatment (yes, no), and ASCVD risk score (continuous). All tests were two sided; *p*‐values < 0.05 were considered significant. All analyses were performed using RStudio (Version 2023.12.0.369, RStudio Team, 2023) [44].

## 3 Results

### 3.1 Patient and Clinical Characteristics

The cohort comprised 300 patients (mean age = 58.9 ± 13.4 years; 42% female) with colon (66%) or rectal (34%) cancer (Table 1). The cohort was racially and ethnically diverse, including individuals who self‐identified as non‐Hispanic White (60%), Hispanic/Latino (18%), Asian (13%), and African American/Black (9%). At the time of diagnosis, 157 individuals were overweight/obese (59%), with a higher proportion in males compared to females (62% vs. 56%). Approximately half the individuals had advanced‐stage CRC at the time of enrollment (III = 34%, IV = 20%). Nearly all patients underwent surgery (93%); most received chemotherapy (62%) and/or radiotherapy (24%).

**TABLE 1:** Patient demographic and clinical characteristics overall and by CAC score at the time of diagnosis.

Variables	Entire cohort (*N* = 300)	Baseline scan^a^ (*N* = 101)		
		CAC = 0 (*N* = 64)	CAC > 0 (*N* = 37)	*p*
Demographic characteristics				
Age	58.9 ± 13.4	51.9 ± 12.2	67.1 ± 12.9	< 0.001
Sex				
Female	127 (42%)	29 (45%)	11 (30%)	0.183
Male	173 (58%)	35 (55%)	26 (70%)	
Race and ethnicity				
Non‐Hispanic White	176 (60%)	40 (63%)	29 (81%)	
Hispanic or Latino	51 (18%)	10 (16%)	0 (0%)	
Asian	37 (13%)	8 (13%)	5 (14%)	0.048
Black or African American	27 (9%)	5 (8%)	2 (5%)	
Other/Unknown	9	1	1	
BMI (kg/m^2^)	26.5 ± 5.13	26.6 ± 5.2	25.5 ± 3.5	0.216
Epicardial fat	98.6 ± 73.5	80.09 ± 60.9	104.2 ± 63.6	0.150
Visceral fat	193.3 ± 149.9	162.1 ± 147.4	219.3 ± 157.3	0.170
Thoracic fat	233.1 ± 161.8	197.7 ± 155.8	237.7 ± 129.9	0.604
Muscle mass	185 ± 74.8	187.7 ± 75.1	177.6 ± 63.8	0.586
Baseline lifestyle characteristics				
Smoking status				
Never	168 (61%)	46 (72%)	20 (54%)	0.043
Former	87 (32%)	17 (27%)	13 (35%)	
Current	21 (8%)	1 (2%)	4 (11%)	
Unknown/missing	24	0	0	
Alcohol use				
Never	69 (26%)	11 (18%)	10 (27%)	0.034
Former	75 (28%)	23 (37%)	5 (14%)	
Current	120 (45%)	28 (45%)	22 (59%)	
Unknown/missing	36	2	0	
History of comorbidities				
Hypertension	111 (37%)	14 (22%)	21 (57%)	< 0.001
Hyperlipidemia	73 (24%)	8 (12%)	19 (51%)	< 0.001
Diabetes mellitus	42 (14%)	5 (8%)	9 (24%)	0.043
Heart failure	11 (4%)	2 (4%)	3 (8%)	0.371
Stroke	12 (5%)	0 (0%)	5 (14%)	0.007
CAD	16 (6%)	1 (2%)	7 (19%)	0.004
MACE	20 (8%)	1 (2%)	8 (22%)	0.001
Medication use				
Statin	37 (12%)	8 (12%)	11 (30%)	0.061
Metformin	15 (5%)	4 (6%)	4 (11%)	0.459
ACE2 inhibitors	6 (2%)	0 (0%)	1 (3%)	0.366
Beta blockers	18 (6%)	2 (3%)	2 (5%)	0.622
Aspirin	15 (5%)	2 (3%)	6 (16%)	0.048
Atherosclerotic CVD risk category (ASCVD)				
Low (0 – < 5%)	8 (3%)	6 (9%)	1 (3%)	
Borderline (5 – < 7.5%)	61 (20%)	10 (16%)	7 (19%)	
Intermediate (7.5 – < 20%)	59 (20%)	10 (16%)	7 (19%)	0.652
High (> 20%)	172 (57%)	38 (59%)	22 (59%)	
ASCVD score^b^	20.93 ± 10.70	20.26 ± 11.25	21.96 ± 13.63	0.541
Tumor characteristics				
Tumor site				
Left‐sided colon	99 (33%)	22 (34%)	15 (41%)	
Right‐sided colon	72 (24%)	12 (19%)	11 (30%)	0.380
Rectal	102 (34%)	21 (33%)	8 (22%)	
Unspecified colon site	27 (9%)	9 (14%)	3 (8%)	
Stage				
0	10 (3%)	2 (3%)	1 (3%)	
I	45 (16%)	4 (6%)	5 (14%)	0.415
II	78 (27%)	17 (27%)	5 (14%)	
III	97 (34%)	19 (31%)	14 (39%)	
IV	56 (20%)	20 (32%)	11 (31%)	
Unknown	14	2	1	
Cancer treatment				
Chemotherapy/Antibody drug				
Yes	186 (62%)	48 (75%)	28 (76%)	1
No	114 (38%)	16 (25%)	9 (24%)	
Radiotherapy				
Yes	70 (24%)	17 (27%)	8 (22%)	0.752
No	227 (76%)	47 (73%)	29 (78%)	
Unknown	3			
Surgery				
Yes	279 (93%)	64 (100%)	37 (100%)	NA
No	21 (7%)	0 (0%)	0 (0%)	

Abbreviations: ACE, angiotensin‐converting enzyme; BMI, body mass index; CAC, coronary artery calcium; MACE, major adverse cardiovascular events.

^a^ A baseline scan is defined as a computed tomography (CT) scan performed before or within six months of a cancer diagnosis.

^b^ Calculated ASCVD score and imputed values using Multiple Imputation by Chained Equations (MICE) method.

### 3.2 Cardiometabolic Risk Factors and MACE

Before CRC diagnosis, a substantial proportion of CRC patients had a history of cardiometabolic disease including hypertension (37%), hyperlipidemia (24%), and DM (14%) (Table 1). A smaller number of patients had a history of HF (4%), stroke (5%), CAD (6%), and/or MACE (8%) before diagnosis. About a quarter of CRC patients underwent TTE imaging (27.0%) or received a cardiologist referral (21.3%) after their diagnosis of CRC. Of patients who had a TTE, 38.9% had repeat imaging and 46.6% of those with follow‐up imaging showed worsening ejection fraction or new diastolic dysfunction following their CRC diagnosis. In addition, several patients developed new or worsening hypertension (7%) and/or hyperlipidemia (6%) after their CRC diagnosis.

### 3.3 CAC at CRC Diagnosis

Among patients with an available CT scan without contrast at baseline (33.6%, *n* = 101/300), over one‐third (36.6%, *n* = 37/101) had CAC (mean = 171.8). As expected, individuals with CAC were older (67.1 vs. 51.9 years, *p* < 0.01) and had a history of hypertension (57% vs. 22%, *p* < 0.01), hyperlipidemia (51% vs. 12%, *p* < 0.01), and DM (24% vs. 8%, *p* < 0.04) compared to those with no radiographic evidence of CAC. Individuals with CAC had nonsignificantly higher epicardial fat (104.2 vs. 80.09, *p* = 0.15), visceral fat (219.3 vs. 162.1, *p* = 0.17), and thoracic fat (237.7 vs. 197.7, *p* = 0.60) compared to those without CAC. ASCVD score was not significantly elevated among those with CAC present at the time of CRC diagnosis (CAC = 0: 20.26 ± 11.25 vs. CAC > 0: 21.96 ± 13.63, *p* = 0.54). Among patients with CAC, 10 (27%) had an Agatston score of 100–400 and 10 (27%%) had a score > 400, indicating evidence of moderate to extensive atherosclerosis, respectively. Among those with CAC > 100, 11 (55.0%) patients were not receiving statins.

### 3.4 Change in CAC After CRC Diagnosis

There were 61 patients (20%) who had at least two imaging scans available at least 1 year apart. Of these patients, 19 (31.1%) had progression of atherosclerosis as evidenced by an increase in CAC after 1 year. Two (3%) patients developed CAC after CRC treatment. When comparing CAC risk groups based on imaging scans from the first and last time points, we found that the proportion of patients with elevated CAC scores (> 100) increased from 22.8% at the first scan to 27.9% at the follow‐up (*p* = 0.22).

### 3.5 CAC and Subsequent MACE

Among the entire cohort, 25% (*n* = 75) of patients experienced MACE after CRC diagnosis (median follow‐up time = 5.3 years (interquartile range [IQR] = 4.1–6.6 years)), including HF exacerbation requiring hospitalization (*n* = 4, 2%), new or worsening HF (*n* = 22, 9%), revascularization procedure required (*n* = 7, 3%), and stroke (*n* = 7, 3%, Table 2). Among the 57 deaths, cancer was the underlying cause in approximately more than half of patients (33 deaths). Most MACE events occurred > 6 months after diagnosis (83%) (median time between MACE and CRC diagnosis =1.88 [IQR = 0.76–3.15]). In the Fine and Gray model adjusted for potential confounders, individuals with CAC > 0 had a significantly increased risk of MACE (HR: 4.79, 95% CI: 1.05–21.75) compared to those without CAC, accounting for cancer‐related deaths as competing events (Table 3).

**TABLE 2:** MACE outcomes by coronary artery calcium score among patients with CRC.

Measurement	Entire cohort (*N* = 300)	Baseline scan^a^ (*N* = 101)		
		CAC = 0 (*N* = 64)	CAC > 1 (*N* = 37)	*p* ^b^
HF exacerbation requiring hospitalization	4 (2%)	0 (0%)	1 (3%)	
Within 6‐month CRC diagnosis	2	0	0	
> 6 month after CRC diagnosis	2	0	1	0.387
Unknown date	0	0	0	
New or worsening HF	22 (9%)	2 (4%)	4 (11%)	
Within 6‐month CRC diagnosis	5	1	1	
> 6 month after CRC diagnosis	15	1	3	0.201
Unknown date	2	0	0	
Revascularization procedure (PCI or CABG)	7 (3%)	1 (2%)	0 (0%)	
Within 6‐month CRC diagnosis	0	0	0	1
> 6 months after CRC diagnosis	5	1	0	
Unknown date	2	0	0	
Stroke	7 (3%)	1 (2%)	1 (6%)	
Within 6‐month CRC diagnosis	3	0	1	
> 6 months after CRC diagnosis	4	1	0	1
Unknown date	0	0	0	
All‐cause mortality	57 (19%)	14 (22%)	14 (38%)	
Within 6‐month CRC diagnosis	3	2	1	0.107
> 6 months after CRC diagnosis	54	12	13	
Unknown date	0	0	0	
MACE^c^	75 (25%)	15 (26%)	17 (47%)	
Within 6‐month CRC diagnosis	11	3	2	0.046
> 6 months after CRC diagnosis	62	12	15	
Unknown date	2	0	0	

Abbreviations: CABG, coronary artery bypass graft; CAC, coronary artery calcium; CRC, colorectal cancer; HF, heart failure; MACE, major adverse cardiovascular events; PCI, percutaneous coronary intervention.

^a^ A baseline scan is defined as a CT scan performed before or within 6 months of a cancer diagnosis.

^b^ Fisher's exact test comparing individuals with and without CAC at baseline.

^c^ MACE defined here as the first event in patients with more than one event.

**TABLE 3:** Association of CAC score at CRC diagnosis with MACE risk using fine and gray competing risks model.

Sub distribution HR accounting for cancer‐related deaths as competing risk						
Measurement	CAC = 0 (*N* = 64)			CAC > 1 (*N* = 37)		
	Number of events	Person‐time at risk	HR (95% CI)	Number of events	Person‐time at risk	HR (95% CI)
Model 1	6	265.97	1.00 (ref)	9	124.08	3.64 (0.99, 13.36)
Model 2	6	265.97	1.00 (ref)	9	124.08	4.79 (1.05, 21.75)

*Note:* Model 1 adjusted for age and Model 2 adjusted for age, sex, tumor site, chemotherapy treatment, and ASCVD risk score.

Abbreviations: CAC, coronary artery calcium; CI, confidence interval; CRC, colorectal cancer; HR, hazard ratio; MACE, major adverse cardiovascular events.

## 4 Discussion

In our prospective cohort study, we show that more than one‐third of patients with CRC have subclinical atherosclerosis with an increase in risk for MACE. We also discovered that 55.0% of patients with moderate or greater severity of atherosclerosis were not treated with statins. To our knowledge, this is the first study to describe the prevalence of subclinical atherosclerosis among CRC patients using CT imaging that was performed for cancer staging. Over the median follow‐up period of approximately 5 years, CAC worsened in 31% of patients and was associated with an increased risk of MACE after adjusting for cancer‐related deaths as a competing risk.

The prevalence of CAC in our cohort of CRC patients was somewhat higher than studies among patients with breast cancer (36.6% vs. 10%) [45] and lower compared to studies in patients with lung cancer (36.6% vs. 60.7%) [46]. In addition, our study found that a small percentage (4.3%) of patients with CAC detected on CT scans had their CAC quantified and reported clinically. Other studies have also shown low rates of clinical reporting for CAC in patients with cancer, ranging from 6.4% to 41.2% [29, 34]. Possible reasons for this underreporting are the focus on detecting metastases on CTs rather than mitigating CVD. Additionally, the lack of emphasis on CAC reporting may be partially attributed to the lack of guidelines advising providers to routinely screen for CAC in CRC patients. The United States Preventive Services Task Force (USPSTF) states that there is currently insufficient evidence to incorporate CAC into traditional risk assessment for CVD in asymptomatic adults.

The lack of reporting of CAC in cancer patients may be a missed opportunity for early intervention of CVD. In our cohort, only 45% of patients with moderate to severe CAC at baseline were receiving statins, which is concurrent with other studies that have shown that many cancer patients with subclinical atherosclerosis are not prescribed preventative medications [30, 31, 34]. This observation is particularly noteworthy in light of the fact that statin treatment has been shown to be associated with favorable outcomes in individuals with CAC > 100 [47]. The AHA guidelines recommend patients of at least borderline ASCVD risk and CAC, particularly moderate–severe, should also receive statins [48]. Not only is underreporting of CAC a potential concern, some studies suggest that even when CAC is reported, aspirin or statin is prescribed only 5% of the time [49]. These findings raise concerns about the suboptimal care of patients with CAC, highlighting a gap in the management of CVD risk in CRC patients.

Currently, the ASCVD risk score estimates absolute rates of CVD events over 10 years and guides implementation of preventive measures such as lifestyle adjustments, statins, and antihypertensive therapies. However, it is important to acknowledge that the ASCVD score, along with other standard risk stratification methods, may under‐ or overestimate risk in certain populations [50, 51]. Notably, it has not been validated in cancer patients [48, 52] and is likely to underestimate risk in those receiving potentially cardiotoxic treatments including chemotherapy, immunotherapy, thoracic radiation therapy, and molecularly targeted agents. Therefore, additional approaches may be useful to optimize risk stratification, and further studies are needed to evaluate the performance of traditional risk assessments plus CAC scores.

In our cohort, we observed a relatively high rate of MACE (25%) among CRC patients after diagnosis. This is in line with prior research that has shown that the risk of MACE is notably elevated in cancer patients compared to the general population [29, 30, 31, 32, 33, 34, 35]. The increased risk can be attributed to factors related to both the prothrombotic state associated with malignancy [53] and the cardiotoxic effects of certain treatment modalities such as chemotherapy, radiation, and immunotherapy [19, 20, 21, 22]. Additionally, issues such as cachexia, persistent inflammation, and cardiotoxic oncometabolites have also been demonstrated to elevate the likelihood of CVD in cancer patients [54]. Patients with cancer also tend to continue to experience a deterioration in cardiovascular risk factors or CVD during cancer treatment, further contributing to the heightened risk of MACE [55], which was also demonstrated in our study.

Nearly more than half of deaths in our cohort were attributed to cancer. These findings are aligned with a past study that showed that CRC patients are more likely to die from cancer within the first 5 years of diagnosis, while CVD becomes the primary cause of mortality after a decade into survivorship [56]. Furthermore, patients with CAC present were more likely to die compared to patients with no CAC present. These findings suggest that there may be a relationship between higher CAC levels and increased mortality in CRC patients. This is in line with other studies that have shown a relationship between CAC and all‐cause mortality in asymptomatic adults [57, 58] and lung cancer patients [46]. The presence of CAC was also associated with a four times higher risk of MACE compared to those without CAC, after accounting for the competing risk of cancer. Other research showed in a general population that those with any CAC had significantly higher long‐term risk of MACE and MI, while severe CAC increased risk for all outcomes including death [59].

There are limitations of our study. Most patients undergo contrast‐enhanced CT imaging for staging and surveillance. This precludes accurate measurement of CAC, which requires noncontrast scans for quantification. Additionally, follow‐up imaging was often unavailable—either never performed, obtained with contrast, or conducted outside our institution—limiting our ability to evaluate CAC progression over time. CAC was also quantified using non‐ECG gated CT scans and could overestimate CAC measurements by visual assessment points to the presence of atherosclerosis in these subjects. We did not observe an association between anticancer therapy and CVD outcomes, which could be attributable to our limited sample size and inability to stratify by specific therapy type. Larger studies are warranted to evaluate the potential additive cardiovascular risks from agents such as immune checkpoint inhibitors, which may accelerate atherosclerosis [60], and VEGF inhibitors, which have been associated with hypertension [61].

The advantages of our study include the systematic collection of data prospectively and cardiometabolic phenotyping using both EMRs and imaging scans. Only a few previously reported studies have examined subclinical atherosclerosis in patients with cancer, and these studies are limited to retrospective collection using EMRs as the primary source of information.

In conclusion, unidentified, subclinical atherosclerosis appears to be highly prevalent in CRC patients. Future research is needed to better understand CVD in this susceptible population, and interventions to reduce cardiovascular morbidity and mortality in this high‐risk population may be warranted.

## Author Contributions

**Julia A. Levy:** data curation (equal), formal analysis (equal), writing – original draft (equal), writing – review and editing (equal). **Jane C. Figueiredo:** formal analysis (equal), funding acquisition (equal), investigation (equal), project administration (equal), resources (equal), supervision (equal), writing – original draft (equal), writing – review and editing (equal). **Elham Kazemian:** data curation (equal), formal analysis (equal), validation (equal), writing – original draft (equal), writing – review and editing (equal). **Cody Ramin:** writing – review and editing (equal). **Nicole C. Loroña:** data curation (equal), formal analysis (equal), supervision (equal), validation (equal), writing – original draft (equal), writing – review and editing (equal). **Maimoona Nadri:** investigation (equal), writing – review and editing (equal). **Jordan O. Gasho:** writing – review and editing (equal). **Katrina D. Silos:** investigation (equal), writing – review and editing (equal). **Andriana P. Nikolova:** investigation (equal), writing – review and editing (equal). **Damini Dey:** investigation (equal), writing – review and editing (equal). **Erin M. Siegel:** investigation (equal), resources (equal), writing – review and editing (equal). **Biljana Gigic:** investigation (equal), resources (equal), writing – review and editing (equal). **Sheetal Hardikar:** investigation (equal), resources (equal), writing – review and editing (equal). **Doratha A. Byrd:** investigation (equal), resources (equal), writing – review and editing (equal). **Adetunji T. Toriola:** investigation (equal), resources (equal), writing – review and editing (equal). **Jennifer Ose:** investigation (equal), resources (equal), writing – review and editing (equal). **Christopher I. Li:** investigation (equal), resources (equal), writing – review and editing (equal). **David Shibata:** investigation (equal), resources (equal), writing – review and editing (equal). **Cornelia M. Ulrich:** funding acquisition (equal), investigation (equal), resources (equal), writing – review and editing (equal). **Balaji K. Tamarappoo:** investigation (equal), resources (equal), writing – review and editing (equal). **Katelyn M. Atkins:** investigation (equal), resources (equal), writing – review and editing (equal).

## Disclosure

The authors have nothing to report.

## Conflicts of Interest

The authors declare no conflicts of interest.

## Supporting information


Data S1.

## Data Availability

The data that support the findings of this study are available from the corresponding author upon request.
