# Supplementary material for: Subclinical Atherosclerosis and Cardiovascular Events Among Patients With Colorectal Cancer
Source: Cancer Med. 2025 May 14;14(10):e70938. doi: 10.1002/cam4.70938 (PMC12076194; doi:10.1002/cam4.70938)
Supplement: Supplementary file 1 — Data S1. [file CAM4-14-e70938-s001.docx]

**Supplemental Materials**

**Subclinical atherosclerosis and cardiovascular events among patients with**

**colorectal cancer**

Levy et al.

**Figure 1:** Consort diagram

**
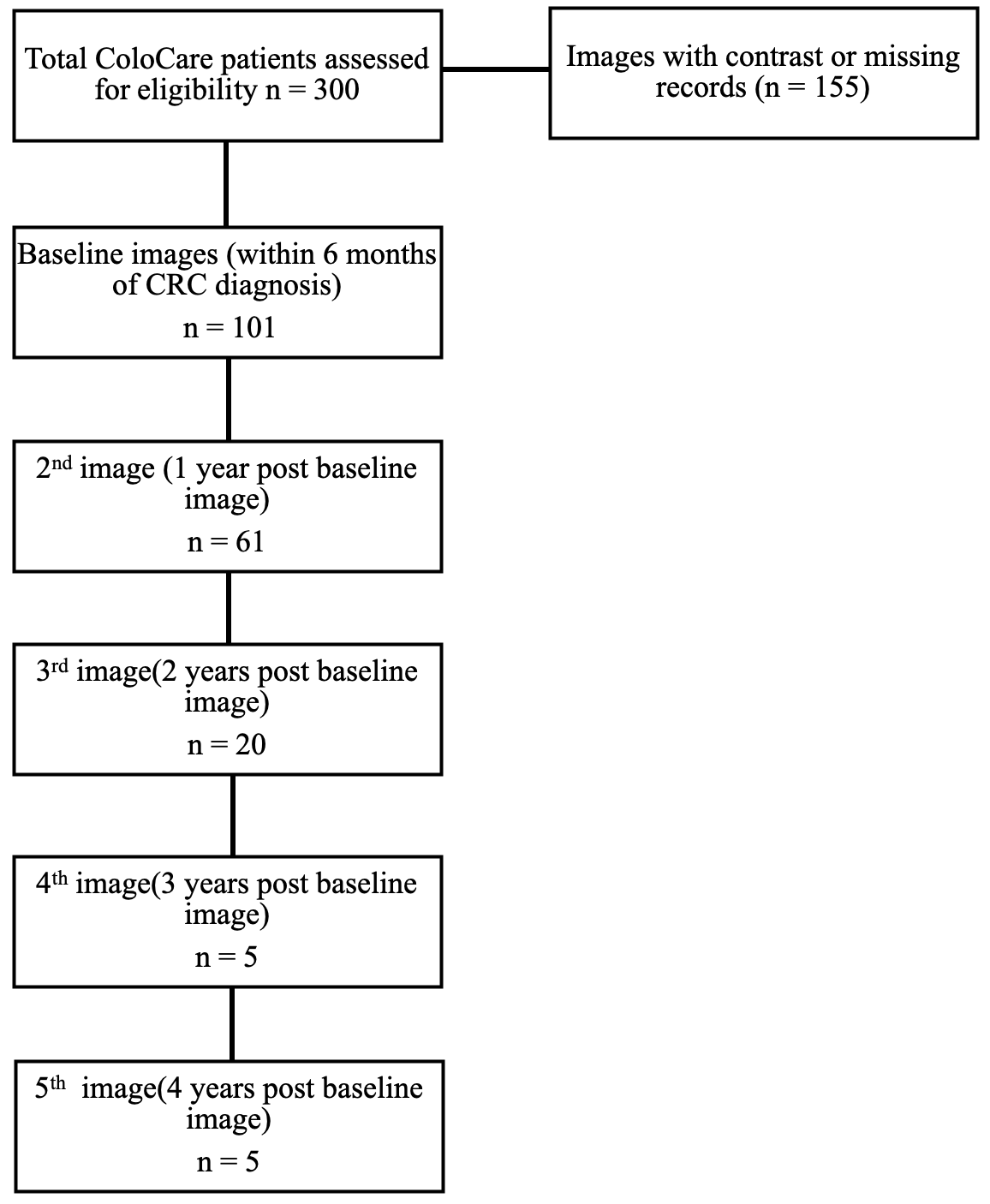
**
